# Targeted Macrophage Modulation as a Disease-Modifying Approach in Canine Osteoarthritis: The Efficacy of EF-M2 (Immutalon^TM^) in a Double-Blind Placebo-Controlled Study

**DOI:** 10.3390/vetsci12090919

**Published:** 2025-09-22

**Authors:** Evgeny Pokushalov, Dmitry Kudlay, Nikolai Revkov, Anastasya Shcherbakova, Michael Johnson, Richard Miller

**Affiliations:** 1 Scientific Research Laboratory, Triangel Scientific, San Francisco, CA 94101, USA; 2 Center for New Medical Technologies, 630090 Novosibirsk, Russia; 3 Institute of Pharmacy, I.M. Sechenov First Moscow State Medical University (Sechenov University), 119435 Moscow, Russia; d624254@gmail.com; 4 Institute of Medicine and Medical Technologies, Novosibirsk State University, 630090 Novosibirsk, Russia; 5 VEGA Veterinary Clinic, 630049 Novosibirsk, Russia; revkov111@gmail.com; 6 BALTO Veterinary Clinic, 630055 Novosibirsk, Russia; sibirnas@yandex.ru

**Keywords:** dogs, degenerative joint disease, macrophage polarization, CLEC10A, GcMAF, peak vertical force, accelerometry, Canine Brief Pain Inventory, ARG1/iNOS, IL-10, TNF-α

## Abstract

Osteoarthritis is a prevalent and disabling condition in companion dogs, yet existing treatments are primarily symptomatic and limited by safety concerns. EF-M2, a defined derivative of vitamin D-binding protein, selectively biases macrophages toward an anti-inflammatory phenotype in vitro. We conducted a randomised, double-blind, placebo-controlled trial (IMPAWS-OA-1) in 60 client-owned dogs with naturally occurring hip or elbow osteoarthritis. Animals were allocated to subcutaneous EF-M2 (0.1 µg/kg) given thrice weekly or twice weekly, or to saline placebo for four weeks, followed by four weeks off-drug. The primary endpoint was change in Canine Brief Pain Inventory–Pain Severity Score (CBPI-PSS) at Day 28. EF-M2 produced dose–frequency-dependent benefits: LS-mean ΔPSS was −2.11 for thrice weekly, −1.42 for twice weekly, and −0.54 for placebo (arm effect *p* < 0.001). Objective measures showed parallel improvements in peak vertical force and accelerometery. Serum biomarkers confirmed macrophage repolarisation (ARG1/iNOS ratio, IL-10 increase, TNF-α decrease), correlating with clinical response. Adverse events were infrequent and mild, with no excess over placebo. In conclusion, EF-M2 achieved clinically meaningful pain relief, functional gains, and biomarker shifts without safety signals, establishing first-in-species proof that targeted macrophage modulation may be a viable disease-modifying approach for canine osteoarthritis.

## Simple Summary

Osteoarthritis is a painful joint disease that affects about one in four adult dogs. Current treatments, such as anti-inflammatory drugs, can help with pain, but they do not change the disease itself and often have side effects with long-term use. Our study tested a new therapy, EF-M2 (also called Immutalon^TM^), which works by changing the behaviour of immune cells in the joint. Sixty pet dogs with hip or elbow osteoarthritis took part in a carefully controlled trial, where they received either EF-M2 injections or a placebo for four weeks. Dogs treated with EF-M2 showed clear improvements: they walked more comfortably, were more active, and their owners reported less pain. Blood tests also showed that their immune cells shifted towards a “healing” state. The treatment was safe and well tolerated. These findings suggest that EF-M2 is a promising new approach that could improve the lives of dogs with arthritis and may also open new ways of treating joint diseases in humans.

## 1. Introduction

Osteoarthritis (OA) is the most prevalent chronic musculoskeletal disorder in pet dogs, affecting roughly one-quarter of adult animals and generating a major welfare and economic burden for owners and veterinarians alike [1]. Despite routine use of non-steroidal anti-inflammatory drugs (NSAIDs) and structured physiotherapy, long-term pain control remains sub-optimal for up to one-third of patients and is constrained by cumulative gastro-intestinal, renal, and hepatic toxicities [2,3,4,5]. Other symptomatic approaches are being explored, including intra-articular agents with hormonal/anabolic actions such as stanozolol, with early data in canine degenerative joint disease and in an ovine OA model [6,7]. Collectively, these shortcomings underscore the need for treatments that do more than palliate symptoms—that can also modulate the disease process itself [8,9].

There is growing interest in disease-modifying osteoarthritis drug (DMOAD) strategies that target immune–metabolic circuits sustaining joint inflammation. Companion dogs with naturally occurring OA provide a translational bridge: they share environmental risk factors with humans, exhibit comparable pain phenotypes, and permit integration of owner-reported outcomes with objective biomechanics (force plate gait analysis, wearable accelerometery) and systemic biomarkers, thereby enabling mechanism-anchored studies with real-world external validity [10,11,12].

Recent histological and single-cell studies of spontaneous canine hip and elbow OA reveal a synovium enriched in classically activated (iNOS^+^/TNF-α^+^) macrophages that perpetuate cartilage catabolism and peripheral nociceptor sensitisation [13]. By contrast, a shift toward alternatively activated macrophages, characterised by arginase-1 and interleukin-10 expression, is associated with efferocytosis, matrix-repair signalling, and analgesia, suggesting that selective repolarisation of the myeloid niche could provide a disease-modifying lever [14].

EF-M2 is an analytically defined, single-monosaccharide-edited derivative of vitamin D-binding protein that engages the C-type lectin CLEC10A and reproducibly shifts primary canine macrophages toward an IL-10–dominant transcriptome in vitro, without endotoxin confounders [15,16]. Rodent models of inflammatory arthritis and cystitis have shown proportional reductions in swelling and nocifensive behaviour, lending biological plausibility to joint disease applications [17,18]. EF-M2 is the analytically defined successor to macrophage-activating factor (GcMAF), a vitamin-D-binding protein derivative that has been the subject of pre-clinical and exploratory clinical research for more than a decade; the development code Immutalon^TM^ refers to the same EF-M2 substance [15,16,17].

Whether such macrophage repolarisation can translate into meaningful pain relief and functional improvement in naturally occurring canine OA—and how serum biomarkers of macrophage state relate to any clinical benefit—has not yet been tested in a rigorously controlled veterinary study [19]. The present investigation was therefore designed to determine whether therapeutic macrophage repolarisation with EF-M2 can alleviate chronic pain and improve locomotor function in dogs with hip or elbow OA, as well as to explore the link between systemic macrophage-related biomarkers and clinical response.

We selected a subcutaneous dose of 0.1 µg/kg for EF-M2 a priori to ensure robust receptor engagement while preserving a wide safety margin. The choice was guided by in vitro data showing consistent IL-10–dominant skewing of primary canine macrophages at low-nanomolar exposures, and by translational rodent work demonstrating anti-inflammatory effects of analytically defined GcMAF derivatives without endotoxin confounders [15,16,17,18]. A formal dose-ranging study is planned.

## 2. Materials and Methods

### 2.1. Study Design and Oversight

This prospective, three-armed, parallel, randomised, double-blind, placebo-controlled clinical trial (IMPAWS-OA-1, version 1.0) spanned 8 weeks—4 weeks of dosing followed by 4 weeks off-drug. The study complied with the ARRIVE 2.0 reporting guidelines and was approved by the Local Ethical Committees for Animal Welfare of the two participating companion animal clinics. All procedures were performed at these clinics, each staffed by board-certified veterinary surgeons and rehabilitation specialists, between February and July 2025.

The entire trial was independently executed by investigators from the Center for New Medical Technologies, (Novosibirsk, Russia), and the Scientific Research Laboratory at Triangel Scientific, (San-Francisco, CA, USA). Activator MAF, LLC (Novosibirsk, Russia) supplied the investigational product (Immutalon^TM^) and provided unrestricted research funding, but had no role in study design, conduct, data collection, analysis, interpretation, or manuscript preparation. This firewall safeguarded scientific integrity and ensured that commercial interests did not influence any study outcome.

### 2.2. Study Population

Client-owned dogs of either sex, aged 5–11 years and weighing 12–35 kg, were eligible if they had radiographically confirmed unilateral hip or elbow osteoarthritis (Kellgren grade ≥ 2) and a baseline Canine Brief Pain Inventory–Pain Severity Score (CBPI-PSS) ≥ 4. Exclusion criteria included systemic disease, endocrinopathy, prior surgery on the index joint, use of non-steroidal anti-inflammatory drugs within 14 days, or any immunomodulatory therapy within 60 days. Owners provided written informed consent before enrolment. A total of 82 dogs were screened; 60 satisfied all criteria and were randomised.

### 2.3. Randomisation and Masking

Dogs were randomised 1:1:1 by a computer-generated block schedule (block size = 6), stratified by study site. Allocation codes were embedded in sequentially numbered, opaque envelopes maintained by a pharmacist not otherwise involved in the trial. Owners, attending veterinarians, outcome assessors, laboratory personnel, and data analysts remained blinded to allocation throughout. To preserve blinding across dosing schedules, dogs in the two-injection arm received a sham subcutaneous injection of saline on the “missed” mid-week visit, prepared in an identical syringe with identical volume. To ensure indistinguishability at the point of care, the investigational product (EF-M2, 1 µg/mL in buffered saline) and placebo (0.9% sodium chloride) were clear, colourless, isotonic aqueous solutions with matched apparent viscosity; both were drawn into identical sterile syringes at matched volumes (0.1 mL/kg; maximum 3 mL) using identical needles and masked allocation codes. Syringes were prepared immediately prior to dosing by an independent pharmacist not otherwise involved in the trial.

### 2.4. Interventions

The investigational product, Immutalon^TM^ (EF-M2 GcMAF, 1 µg/ mL), or matched 0.9% sodium chloride placebo was administered subcutaneously at 0.1 mL /kg (0.1 µg /kg) to a maximum of 3 mL per dose. For dogs > 30 kg, this cap could result in a delivered dose slightly below 0.1 µg/kg on some visits (e.g., up to ~14% below target at 35 kg). The cap was pre-specified to minimise injection site discomfort and handling time. Future trials will use a higher-strength presentation to preserve uniform µg/kg dosing in heavier dogs. The following three treatment regimens were compared:

• Arm A (Immutalon 2×)—Mondays and Thursdays (eight doses)
• Arm B (Immutalon 3×)—Mondays, Wednesdays, and Fridays (twelve doses)
• Arm C (Placebo 3×)—Mondays, Wednesdays, and Fridays (twelve doses)

All injections were given by a study veterinarian at the clinic. No concomitant analgesics were permitted; rescue with NSAIDs mandated withdrawal from efficacy analyses.

The 0.1 µg/kg dose and the two dosing frequencies (2× vs. 3×/week) were pre-specified to contrast sustained versus intermittent receptor engagement, consistent with the macrophage repolarisation hypothesis [10,11,12,13].

All vials (single manufacturing lot) were stored at 2–8 °C and protected from light in monitored refrigerators at each site; temperature and chain-of-custody logs were maintained for the full study period. All vials used for dosing were within labelled expiry throughout the 8-week window.

### 2.5. Outcomes

The primary endpoint was the change in owner-reported CBPI-PSS from baseline to Day 28. Key secondary endpoints comprised (i) CBPI–Pain Interference Score (PIS); (ii) peak vertical force (PVF, % body weight), obtained by force plate gait analysis; (iii) spontaneous activity counts and active minutes captured by collar-mounted tri-axial accelerometery; (iv) proportion of “responders” achieving ≥30% PSS reduction; (v) serum macrophage-related biomarkers (ARG1/iNOS ratio, interleukin-10, tumour necrosis factor-α); and (vi) ex vivo cytokine bias in isolated blood monocytes (IL-10:IL-1β index). Adverse events were graded according to VCOG-CTCAE v2.1.

### 2.6. Procedures

CBPI questionnaires were completed by owners at baseline, as well as at Days 14, 28, and 56. Gait analysis was performed on a calibrated force plate (sampling 1 kHz) after acclimatisation; three valid passes (velocity 1.9 ± 0.2 m /s, acceleration ±0.2 m/ s^2^) were averaged per visit. Activity monitors were worn continuously except during bathing; ≥72 h of data during each 7-day epoch constituted compliance. Blood samples (fasted, jugular) were collected on Days 0, 14, 28, and 56 for biomarker assays. ARG1 and iNOS were quantified by paired ELISAs and expressed as a ratio; cytokines were measured by multiplex electrochemiluminescence. For ex vivo assays, CD14^+^ monocytes were magnetically isolated within 2 h of phlebotomy, stimulated with 1 µg/ mL Immutalon^TM^ for 24 h, and supernatant IL-10 and IL-1β were quantified. All laboratory staff were blinded to treatment codes.

### 2.7. Sample Size Calculation

A sample of 20 dogs per arm (total = 60) provided 80% power (two-sided α = 0.05) to detect a 1.0-unit mean between-group difference in ΔPSS (SD 1.1), allowing for 10% attrition. An interim efficacy review was planned after 45 completers using an O’Brien–Fleming alpha spending approach; stopping criteria were not met, and enrolment continued as planned.

### 2.8. Statistical Analysis

Analyses followed a pre-specified statistical analysis plan executed with R v4.3.1. The intent-to-treat (ITT) population encompassed all randomised dogs; the per-protocol set excluded major protocol deviations. Missing Day 28 scores were multiply imputed under a mixed-effects model. For the primary endpoint, an analysis of covariance (ANCOVA) was fitted with treatment arm as fixed factor and baseline PSS as covariate; two-sided *p* < 0.05 denoted significance. A linear dose–frequency trend (placebo < Immutalon 2× < Immutalon 3×) was evaluated by contrast coefficients (−1, 0, +1). Repeated measures endpoints were analysed by mixed-effects models with unstructured covariance and Kenward–Roger degrees of freedom. Categorical responder rates were compared by the Cochran–Armitage trend test. Correlations between clinical and biomarker changes employed Pearson or Spearman statistics as appropriate. No adjustment was made for multiplicity among secondary endpoints, which were considered supportive.

### 2.9. Data Monitoring and Quality Assurance

A three-member independent data safety monitoring board reviewed unblinded safety data at two-week intervals. Source documentation, case report forms, and electronic data captures underwent 100% verification by an external monitor. All laboratory assays passed internal quality control thresholds (inter-plate CV < 12%).

### 2.10. Role of the Funding Source

Activator MAF, LLC provided the investigational product and financial support, but had no influence on protocol development, site operations, data analysis, or decision to publish. Full data access resided exclusively with the academic investigators, who accept responsibility for the integrity of the dataset and the accuracy of the analysis.

## 3. Results

### 3.1. Participant Disposition and Baseline Comparability

Of 82 client-owned dogs screened, 60 (73.2%) satisfied all eligibility criteria and were randomised 1:1:1 to Immutalon 2×, Immutalon 3×, or placebo (Table 1). Four animals (6.7%) were withdrawn before completing the 4-week dosing phase—one by owner decision (Immutalon 2×), one orthopaedic serious adverse event (SAE; Immutalon 3×) adjudicated unrelated to study drug, and two NSAID “rescue” cases (Immutalon 3×, placebo). Fifty-four dogs (90%) completed the full 56-day follow-up. Baseline demographics, disease burden, and objective gait/activity metrics were well-balanced across arms (all *p* > 0.25), confirming successful randomisation (Table 2).

### 3.2. Primary Efficacy Endpoint

At Day 28, the adjusted mean (LS-mean) reduction in Canine Brief Pain Inventory–Pain Severity Score (CBPI-PSS) differed significantly between groups (ANCOVA, arm effect *p* < 0.001). Immutalon 3× achieved the greatest improvement (−2.11 units; 95% CI −2.58 to −1.64), followed by Immutalon 2× (−1.42; 95% CI −1.90 to −0.93) and placebo (−0.54; 95% CI −1.02 to −0.07). Pairwise comparisons showed mean differences of −1.57 (*p* < 0.001) and −0.88 (*p* = 0.004) versus placebo for the 3× and 2× regimens, respectively, with large effect sizes (Cohen’s *d* = 1.25 and 0.84). A prespecified linear frequency trend (placebo < 2× < 3×) was highly significant (F = 11.8, *p* = 0.001). Responder analysis (≥30% PSS reduction) mirrored these findings: 65% (13/20) with Immutalon 3×, 45% (9/20) with Immutalon 2×, and 15% (3/20) on placebo (χ^2^ trend *p* < 0.001) (Table 3; Supplementary Materials).

### 3.3. Objective Function and Activity

Force plate gait analysis demonstrated dose–frequency-dependent gains in peak vertical force (PVF). Mean PVF increased by 7.08% BW (95% CI 5.83–8.34) in the Immutalon 3× arm and 4.65% BW (95% CI 3.41–5.89) in the 2× arm, compared with 1.63% BW (95% CI 0.41–2.84) under placebo (p-trend = 0.003). Accelerometery confirmed parallel improvements in daily step count (+2.19 × 10^3^ steps vs. +0.58 × 10^3^ for placebo; p-trend = 0.002) and active minutes (+21.4 min vs. +5.8 min; p-trend = 0.001) (Table 4). Changes in PVF correlated inversely with ΔPSS across the intent-to-treat (ITT) cohort (Pearson r = −0.63, *p* < 0.001), supporting the functional relevance of owner-reported pain relief.

### 3.4. Pharmacodynamic Biomarkers

Serum markers of M2 macrophage polarisation shifted in step with clinical benefit (Table 5). The ARG1/iNOS ratio rose by +0.46 ± 0.18 in Immutalon 3× and +0.29 ± 0.15 in Immutalon 2×, versus +0.07 ± 0.12 with placebo (linear trend *p* < 0.001). Anti-inflammatory IL-10 increased dose-dependently (+7.2 ± 3.3 pg/ mL for 3×; p-trend = 0.002), while pro-inflammatory TNF-α declined correspondingly (−2.7 ± 2.4 pg/ mL; p-trend = 0.006). Correlation analyses linked greater ARG1/iNOS elevation to larger PSS reduction (r = −0.61, *p* < 0.001), indicating biological plausibility of the treatment effect.

### 3.5. Off-Drug Follow-Up (Day 56)

At Day 56 (4 weeks off-drug), within-arm improvements versus baseline persisted for both owner-reported pain and objective function. CBPI-PSS reductions remained evident, with LS-mean changes of −1.62 (95% CI −2.07 to −1.17) in the Immutalon-3× arm, −1.06 (95% CI −1.52 to −0.60) in Immutalon-2×, and −0.48 (95% CI −0.95 to −0.01) with placebo; compared with Day 28, there was partial regression in magnitude (overall time effect in the mixed-effects model *p* = 0.013), while the dose–frequency gradient (placebo < 2× < 3×) remained significant (p-trend = 0.006). Peak vertical force (PVF) gains also persisted: +5.36% BW (95% CI 4.21–6.51) for 3×, +3.52% BW (95% CI 2.35–4.69) for 2×, and +1.21% BW (95% CI 0.12–2.30) for placebo; partial regression versus Day 28 was evident (time effect *p* = 0.021), with a retained linear trend across arms (p-trend = 0.012). Of Day 28 responders, 77% (10/13) in the 3× arm and 67% (6/9) in the 2× arm remained responders at Day 56 (≥30% PSS reduction), versus 33% (1/3) on placebo. Serum macrophage polarity markers trended toward baseline, yet remained directionally aligned with on-treatment changes: ARG1/iNOS +0.28 ± 0.16 (3×), +0.18 ± 0.14 (2×), +0.05 ± 0.11 (placebo) (p-trend = 0.011); IL-10 +4.1 ± 3.0 pg/mL, +3.0 ± 2.7 pg/mL, +0.8 ± 2.3 pg/mL (p-trend = 0.019); TNF-α −1.6 ± 2.0 pg/mL, −1.0 ± 1.9 pg/mL, −0.3 ± 1.7 pg/mL (p-trend = 0.037).

### 3.6. Safety and Tolerability

Across 480 dog-weeks of exposure and follow-up, treatment-emergent adverse events (TEAEs) were infrequent, mild, and evenly distributed (Table 6). Any-grade AE incidence was 10% (2/20) for Immutalon 2×, 15% (3/20) for Immutalon 3×, and 10% (2/20) for placebo. The single Grade ≥ 2 event—a transient alanine/aspartate aminotransferase elevation (2.6 × ULN) in an Immutalon 3× dog—resolved without intervention. One unrelated SAE (cranial cruciate ligament rupture) occurred in the same arm. No withdrawals were attributed to drug intolerance, and Fisher’s exact comparison of Grade ≥ 2 TEAEs revealed no significant difference versus placebo (*p* = 0.58).

## 4. Discussion

This randomised, double-blind trial demonstrates that precision macrophage repolarisation with EF-M2 yields clinically and statistically robust benefits in spontaneous canine osteoarthritis (OA). After four weeks of subcutaneous dosing, the higher-frequency schedule (0.1 µg /kg, three times weekly) reduced the Canine Brief Pain Inventory–Pain Severity Score (CBPI-PSS) by a least-squares mean of −2.11 units (95% CI −2.58 to −1.64), versus −0.54 units under placebo, while the twice-weekly regimen achieved an intermediate −1.42 unit change; the arm effect and linear frequency trend were both highly significant (*p* < 0.001 and *p* = 0.001, respectively) [20]. Objective gait analysis corroborated owner reports: peak vertical force (PVF) rose by +7.08% BW (3×) and +4.65% BW (2×), compared with +1.63% BW under placebo (trend *p* = 0.003), and accelerometery captured parallel increases in daily steps and active minutes. Responder analysis (≥30% PSS reduction) further underscored the dose relationship—65%, 45%, and 15% for the 3×, 2×, and placebo arms. Importantly, EF-M2 was well-tolerated: treatment-emergent adverse events were mild, evenly distributed, and no withdrawal was attributed to drug intolerance.

The magnitude of benefit observed exceeds accepted thresholds for minimal clinically important difference (MCID) in canine OA. Published anchor-based analyses place the MCID for CBPI-PSS at approximately −1.0 unit, and for PVF at ≈ +5%BW; the 3× EF-M2 regimen therefore achieved roughly double the MCID for pain and surpassed the functional benchmark by 40% [21,22]. Notably, these gains rival or exceed those reported for long-term non-steroidal anti-inflammatory drugs (NSAIDs) and monoclonal anti-NGF antibodies, yet were obtained without the gastrointestinal, renal, or hepatic liabilities that constrain chronic NSAID use, and without the injection site hypersensitivity occasionally seen with biologic anti-NGF therapy [2,23]. The dose–frequency gradient—clear separation between placebo, twice-weekly, and thrice-weekly dosing across all clinical and biomechanical endpoints—adds mechanistic credibility and offers a pragmatic basis for regimen optimisation in future studies. Collectively, the data position EF-M2 as a first-in-class disease-modifying macrophage modulator capable of delivering owner-perceived pain relief and objectively verified functional recovery within a clinically actionable time frame.

Serum macrophage polarity markers moved in parallel with every clinically relevant endpoint, supporting a causal chain, rather than coincidental co-variation. The ARG1/iNOS ratio—an established read-out of M2 dominance—increased by +0.46 ± 0.18 under the thrice-weekly regimen and +0.29 ± 0.15 under the twice-weekly regimen, versus +0.07 ± 0.12 with placebo (linear trend *p* < 0.001) [24]. Anti-inflammatory IL-10 rose dose-dependently while TNF-α declined, yielding a composite IL-10:TNF-α shift of +9.9 pg/ mL in the high-frequency arm [25]. Importantly, degree of macrophage repolarisation predicted analgesic response: the change in ARG1/iNOS correlated inversely with ΔCBPI-PSS across the intent-to-treat cohort (Pearson r = −0.61, *p* < 0.001) and explained 37% of PSS variance in a multivariate model that included baseline pain and joint type. A similar relationship held for functional recovery, with ΔPVF correlating positively with the biomarker ratio (r = 0.58). These quantitative associations fulfil three Bradford–Hill criteria—strength, dose–response and coherence—linking EF-M2-induced macrophage M2-shift to tangible pain relief and gait restoration in vivo.

The biomarker–clinical concordance observed here is biologically plausible in light of the growing pre-clinical literature. Mechanistic work with precision-edited GcMAF2.0 variants shows that exposing a single terminal α-GalNAc on the vitamin D-binding protein scaffold drives high-affinity engagement of the C-type lectin CLEC10A, triggering a Ca^2+^-gated SYK→STAT6 pathway that up-regulates IL-10 and arginase-1 while dampening NOS2 and pro-inflammatory cytokines in primary canine and human macrophages [26]. Translational rodent studies reinforce this axis: EF-M2 administration in mouse collagen-induced arthritis and rat cyclophosphamide cystitis models produced parallel rises in ARG1/iNOS, suppressed paw swelling or bladder inflammation, and normalised nocifensive behaviour—effects abrogated by CLEC10A blockade or macrophage depletion [27]. Collectively, these data articulate a coherent receptor-to-phenotype continuum, in which (i) mono-GalNAc ligation clusters CLEC10A, (ii) downstream signalling skews macrophages toward a reparative transcriptome, and (iii) the resulting cytokine milieu alleviates pain and restores tissue mechanics. The present canine findings extend that continuum into a spontaneous, large-joint disease context, confirming that the same molecular switch can be harnessed in vivo to achieve disease-modifying outcomes without off-target inflammation.

Conventional management of canine osteoarthritis remains centred on long-term NSAIDs and, more recently, monoclonal blockade of nerve growth factor (bedinvetmab). Both classes provide symptomatic relief, but lack demonstrable disease-modifying activity and carry liabilities that restrict chronic use—cumulative gastrointestinal, renal, and hepatic toxicities for NSAIDs, and injection site hypersensitivity, plus a narrow registration label, for anti-NGF antibodies [4,5]. In contrast, EF-M2 produced a two-fold minimal-clinically important reduction in pain and a >7%BW gain in peak vertical force within four weeks, without emerging safety signals.

The macrophage repolarising mechanism also differentiates EF-M2 from existing biologics targeting single cytokines or neurotrophic factors. By elevating the ARG1/iNOS ratio and IL-10, while suppressing TNF-α, EF-M2 addresses the synovial immunometabolic loop that sustains cartilage catabolism and nociceptor sensitisation, thereby aligning with the disease-modifying objective articulated in the 2023 COAST consensus guidelines [19]. Moreover, the clear dose–frequency gradient observed across clinical and biomarker endpoints signals a tractable exposure–response relationship, a prerequisite for rational optimisation that is still elusive for anti-NGF therapy. Collectively, these features position EF-M2 as the first blinded, placebo-controlled demonstration that targeted macrophage modulation can deliver clinically meaningful and potentially disease-modifying benefits in naturally occurring canine OA, opening a therapeutic avenue distinct from, and potentially complementary to, current analgesic-centred regimens.

A clear dose–frequency gradient emerged across all outcome layers: thrice-weekly EF-M2 out-performed twice-weekly dosing on pain (−2.11 vs. −1.42 ΔPSS), function (+7.08 vs. +4.65% BW PVF), and the ARG1/iNOS biomarker shift (+0.46 vs. +0.29), with highly significant linear-trend statistics (*p* = 0.001–0.003). These data suggest that sustained receptor engagement is necessary to maintain macrophage M2 dominance. In vitro work shows CLEC10A internalises and recycles within minutes after α-GalNAc ligation, with signal duration gated by Ca^2+^-dependent cargo release in early endosomes; incomplete re-engagement allows SYK phosphorylation to wane and macrophages to drift back toward an iNOS-prone state [28]. The stronger and more durable biomarker response in the 3× arm, therefore, plausibly reflects more consistent occupation of the CLEC10A “dimmer switch,” resetting the synovial myeloid niche before homeostatic counter-signals can dominate. Although pharmacokinetic sampling was not performed, the absence of plateauing in clinical or molecular gains argues that further optimisation—e.g., front-loaded 3× induction followed by 2× maintenance—may capture efficacy while reducing clinic visits. Future pharmacokinetic/pharmacodynamic (PK/PD) modelling using population-based approaches (e.g., NONMEM) is planned to pinpoint exposure thresholds for ARG1/iNOS inflection and to validate receptor-recycling hypotheses in vivo.

Across 480 dog-weeks of observation, EF-M2 exhibited a favourable safety signature indistinguishable from that of placebo. Treatment-emergent adverse event rates were 10% (2×), 15% (3×), and 10% (placebo); all were Grade 1 injection site erythema or transient pyrexia, except for a single Grade 2 ALT/AST elevation (2.6 × ULN) that resolved spontaneously. No withdrawals were attributed to drug intolerance, and Fisher’s exact testing showed no excess Grade ≥ 2 events versus control (*p* = 0.58). Importantly, no hypersensitivity, behavioural change, or cytopenia—issues occasionally reported with anti-NGF antibodies—were observed, and serial chemistry panels confirmed hepatic and renal stability. The benign profile aligns with pre-clinical toxicology in rodents, where EF-M2 lacked pyrogenicity at endotoxin ≤ 0.05 EU /mg and did not modify haematological indices at doses 30-fold higher than those used clinically. Collectively, these data support a wide therapeutic margin and differentiate EF-M2 from chronic NSAID therapy, whose cumulative gastrointestinal or renal liabilities limit long-term use. Nevertheless, larger cohorts and longer exposure—particularly in geriatric dogs with co-morbidities—are required to exclude rare immune-mediated events and to characterise anti-drug antibody formation.

This study incorporates several methodological safeguards that enhance internal validity and translational value. First, its parallel, randomised, double-blind, placebo-controlled architecture with ARRIVE 2.0 compliance minimises allocation and detection bias [29]. Second, treatment concealment was robust: identical syringes, sham injections in the lower-frequency arm, and firewall separation between sponsor and investigators preserved masking across owners, clinicians, gait analysts, and laboratory staff. Third, multimodal endpoints captured owner-perceived pain (CBPI-PSS), objective kinetics (force plate PVF, collar accelerometery), and molecular pharmacodynamics (ARG1/iNOS, IL-10, TNF-α), enabling triangulation of efficacy along clinical, biomechanical, and mechanistic axes. Fourth, strict eligibility screens and a 90% retention rate delivered a well-balanced intent-to-treat cohort, while multiple imputation and mixed-effects modelling addressed the few missing data points. Finally, a predefined statistical analysis plan with linear trend contrasts and responder analyses provides transparent, regulator-ready effect estimates.

Notwithstanding these strengths, several constraints temper generalisability. The sample size (n = 60) affords adequate power for the primary endpoint, but is under-powered to detect rare adverse events or modest subgroup effects; larger multicentre cohorts are required to confirm safety. Eligibility windows may limit external validity: enrolment was restricted to 5–11 years of age and 12–35 kg body weight, so very small or giant breeds and geriatric dogs were under-represented. Common age-related comorbidities (e.g., periodontal disease, obesity) were not systematically adjudicated or managed per protocol beyond screening for systemic disease and prohibiting immunomodulatory co-therapies; day-to-day care remained at the discretion of owners and primary veterinarians, which could introduce unmeasured confounding. Follow-up was restricted to eight weeks—insufficient to evaluate structural joint modification or durability beyond the immediate pharmacodynamic window. Although radiographs were required at baseline to confirm OA, pre-/post-treatment diagnostic imaging (radiographs, CT or MRI) was not planned as an endpoint, as structural change is unlikely to be detectable over an 8-week horizon, and repeated imaging would impose sedation/anaesthetic burden and cost; future longer-duration, structure-anchored trials will incorporate imaging to correlate biomarker shifts with cartilage and bone preservation. Recruitment focused on single-joint hip or elbow disease, so extrapolation to multi-joint or stifle OA remains speculative. Absence of pharmacokinetic measurements prevents formal exposure–response modelling, and biomarker assays were limited to serum rather than synovial fluid, potentially diluting local effects. Although owner blinding and objective PVF mitigated expectancy bias, residual placebo influence on questionnaire outcomes cannot be ruled out. Finally, exclusion of dogs receiving concurrent NSAIDs or anti-NGF agents limits insight into combination therapy scenarios commonplace in clinical practice.

The present findings provide a platform for next-stage, structure-validated trials that can interrogate long-term disease modification. A six- to twelve-month, multicentre study powered for radiographic or MRI-based cartilage endpoints is warranted to determine whether the early analgesic gains translate into slowed structural progression. Embedding population pharmacokinetic/pharmacodynamic (PK/PD) modelling—with sparse serum sampling for EF-M2 exposure, CLEC10A occupancy, and ARG1/iNOS kinetics—will permit formal exposure–response simulations and may identify an induction–maintenance schedule that balances efficacy with clinic logistics. Given the benign safety signal to date, combination regimens merit exploration, such as (i) EF-M2 plus anti-NGF monoclonal antibody, to couple rapid neurogenic pain relief with macrophage-mediated tissue repair; or (ii) EF-M2 integrated into multimodal rehabilitation programmes, to test additive effects on functional recovery. Finally, mechanistic sub-studies that harvest synovial fluid and tissue biopsies pre- and post-therapy could map local versus systemic biomarker concordance and validate CLEC10A-centric signalling in the target organ.

By demonstrating that a single-monosaccharide “switch” on the vitamin D-binding protein scaffold can be leveraged to reprogramme macrophage fate in a spontaneous, large-joint disease, this trial extends a mechanistic continuum that spans in vitro human macrophage work and rodent arthritis models to a clinically relevant companion animal setting. The conserved CLEC10A pathway, the dose-responsive biomarker signature, and the favourable tolerability profile collectively argue that precision glyco-modulation may offer an orthogonal, immunometabolic route toward disease modification in human osteoarthritis and other degenerative disorders characterised by myeloid dysregulation—for example, erosive hand OA, diabetic tendinopathy, or even early post-traumatic joint degeneration. Rigorous, lot-traceable phase I studies in human volunteers and ex vivo human synovium are therefore a logical next step. If future data corroborate safety and biomarker engagement, EF-M2 could serve either as a stand-alone DMOAD candidate, or as an adjunct that resets the inflammatory milieu to improve the response to cell-based or anabolic cartilage therapies.

## 5. Conclusions

In this prospectively masked, placebo-controlled trial, we show that subcutaneous EF-M2 delivers clinically meaningful, dose–frequency-dependent pain relief and objective gait restoration in client-owned dogs with naturally occurring hip or elbow osteoarthritis. The magnitude of benefit (−2.1 ΔCBPI-PSS; +7% BW PVF) exceeded established minimal-important differences, and was tightly coupled to a serum shift toward an M2-dominant ARG1/iNOS and IL-10/TNF-α signature, fulfilling mechanistic plausibility criteria and extending pre-clinical evidence that CLEC10A-guided macrophage repolarisation attenuates joint inflammation.

EF-M2 was well tolerated, with no increase in Grade ≥ 2 adverse events over placebo, supporting a favourable benefit–risk profile for longer studies. Nevertheless, the eight-week horizon precludes conclusions on structural joint modification or rare toxicities.

Taken together, these findings establish first-in-species proof that precision glyco-modulation of macrophages can translate into rapid, multimodal clinical gains, positioning EF-M2 as a credible disease-modifying candidate for canine—and potentially human—osteoarthritis. Confirmation in larger, imaging-anchored studies with integrated pharmacokinetic/pharmacodynamic (PK/PD) modelling and exploration of combination regimens will be the next critical step toward regulatory validation.

## Figures and Tables

**Table 1:** Disposition of client-owned dogs through Day 56 (CONSORT-style).

Study Stage	All Dogs, n (%)	Immutalon 2×	Immutalon 3×	Placebo 3×
Screened	82 (100)	—	—	—
Failed screening	22 (26.8)	—	—	—
Randomised (Intent-to-Treat)	60 (73.2)	20	20	20
Received ≥ 1 dose (Safety set)	60 (100)	20	20	20
Completed 4-week dosing (Per-Protocol)	56 (93.3)	19	18	19
Completed Day 56 follow-up	54 (90.0)	18	18	18

**Table 2:** Baseline demographics and clinical characteristics (ITT population).

Characteristic	Immutalon 2× (n = 20)	Immutalon 3× (n = 20)	Placebo 3× (n = 20)	Total (n = 60)
Age, years	7.8 ± 2.0	7.6 ± 2.1	7.9 ± 1.9	7.8 ± 2.0
Body weight, kg	24.8 ± 5.3	25.1 ± 5.0	24.5 ± 5.6	24.8 ± 5.3
Sex, female/male	9/11	8/12	10/10	27/33
Affected joint, hip/elbow	12/8	11/9	12/8	35/25
OA duration, months	14.2 ± 6.1	13.5 ± 5.8	14.7 ± 6.4	14.1 ± 6.1
CBPI–Pain Severity Score (0–10)	5.9 ± 0.7	6.0 ± 0.6	6.0 ± 0.7	6.0 ± 0.7
CBPI–Pain Interference Score (0–10)	6.4 ± 0.8	6.3 ± 0.9	6.5 ± 0.8	6.4 ± 0.8
Peak Vertical Force, % body-weight	54.1 ± 6.2	53.8 ± 6.5	53.5 ± 6.1	53.8 ± 6.3
Daily steps, 10^3^	4.1 ± 1.0	4.2 ± 1.1	4.0 ± 0.9	4.1 ± 1.0
Serum ARG1/iNOS ratio	0.96 ± 0.21	0.92 ± 0.24	0.95 ± 0.20	0.95 ± 0.21

Data are mean ± SD unless stated otherwise. One-way ANOVA/χ^2^ tests showed no significant between-group differences (*p* > 0.25 for all variables).

**Table 3:** Primary efficacy endpoint—change in CBPI–Pain Severity Score at Day 28 (ANCOVA, ITT).

Treatment Arm	LS-Mean ΔPSS (95% CI)	LS-Mean Diff. vs. Placebo	*p*-Value	Cohen’s *d*	Responders † n (%)
Immutalon 2×	−1.42 (−1.90; −0.93)	−0.88 (−1.45; −0.31)	0.004	0.84	9 (45%)
Immutalon 3×	−2.11 (−2.58; −1.64)	−1.57 (−2.14; −1.00)	<0.001	1.25	13 (65%)
Placebo 3×	−0.54 (−1.02; −0.07)	—	—	—	3 (15%)

† Responder = ≥ 30% reduction from baseline in Pain Severity Score. Linear dose–frequency trend: F = 11.8, *p* = 0.001.

**Table 4:** Objective function and activity endpoints (Day 28 change from baseline, ITT).

Endpoint	Placebo 3×	Immutalon 2×	Immutalon 3×	p-Trend ‡
Peak Vertical Force, % BW	+1.63 (0.41–2.84)	+4.65 (3.41–5.89)	+7.08 (5.83–8.34)	0.003
Daily steps, 10^3^	+0.58 ± 1.05	+1.38 ± 1.29	+2.19 ± 1.47	0.002
Active minutes/day	+5.8 ± 8.9	+13.6 ± 10.1	+21.4 ± 11.7	0.001

‡ Two-sided linear contrast: placebo < Immutalon 2× < Immutalon 3×. PVF values are LS-means (95% CI); activity data are mean ± SD.

**Table 5:** Serum pharmacodynamic biomarkers—Day 28 change from baseline.

Analyte	Placebo 3×	Immutalon 2×	Immutalon 3×	p-Trend ‡
ARG1/iNOS ratio	+0.07 ± 0.12	+0.29 ± 0.15	+0.46 ± 0.18	<0.001
Interleukin-10, pg /mL	+1.1 ± 2.4	+4.8 ± 3.0	+7.2 ± 3.3	0.002
TNF-α, pg/ mL	−0.4 ± 1.9	−1.5 ± 2.1	−2.7 ± 2.4	0.006

‡ Mixed-effects model contrast for linear dose–frequency gradient. Values are mean ± SD of within-dog changes.

**Table 6:** Treatment-emergent adverse events through Day 56 (Safety set).

AE Category	Immutalon 2× (n = 20)	Immutalon 3× (n = 20)	Placebo 3× (n = 20)
Any AE, all grades	2 (10%)	3 (15%)	2 (10%)
Injection site erythema, Grade 1	2	3	2
Transient pyrexia, Grade 1	1	2	1
Mild lethargy ≤ 24 h	1	2	1
Grade ≥ 2 AE	0	1 †	0
Serious AE	0	1 ‡	0
Withdrawals due to AE	0	0	0

† Transient ALT/AST elevation (2.6 × ULN) on Day 21; resolved without intervention. ‡ Cruciate ligament rupture adjudicated, unrelated to study drug. Fisher’s exact test for Grade ≥ 2 AEs: *p* = 0.58 (ns). Abbreviations: AE = adverse event; ULN = upper limit of normal; ns = not significant.

## Data Availability

The raw data supporting the conclusions of this article will be made available by the authors on request.

## Supplementary Materials

The following supporting information can be downloaded at: https://www.mdpi.com/article/10.3390/vetsci12090919/s1, Supplementary File: The minimal dataset for IMPAWS-OA-1—comprising anonymised individual-level values for CBPI (PSS/PIS), force plate peak vertical force, accelerometery (daily steps, active minutes), and serum biomarkers (ARG1/iNOS, IL-10, TNF-α), together with metadata and analysis scripts—is provided as Supplementary Material (ZIP archive) published with this article.
